# A Sensor Probe with Active and Passive Humidity Management for In Situ Soil CO_2_ Monitoring

**DOI:** 10.3390/s24186034

**Published:** 2024-09-18

**Authors:** Jacob F. Anderson, David P. Huber, Owen A. Walsh

**Affiliations:** 1 Department of Geosciences, Boise State University, Boise, ID 83725, USA; owenwalsh@u.boisestate.edu; 2 Earth, Environmental, and Resource Sciences, University of Texas at El Paso, El Paso, TX 79902, USA; dphuber@utep.edu

**Keywords:** soil, CO_2_, IRGA, 3D print

## Abstract

Soil CO_2_ concentration and flux measurements are important in diverse fields, including geoscience, climate science, soil ecology, and agriculture. However, practitioners in these fields face difficulties with existing soil CO_2_ gas probes, which have had problems with high costs and frequent failures when deployed. Confronted with a recent research project’s need for long-term in-soil CO_2_ monitoring at a large number of sites in harsh environmental conditions, we developed our own CO_2_ logging system to reduce expense and avoid the expected failures of commercial instruments. Our newly developed soil probes overcome the central challenge of soil gas probes—surviving continuous exposure to soil moisture while remaining open to soil gases—via three approaches: a 3D printed housing (economical for small-scale production) following design principles that correct the usual water permeability flaw of 3D printed materials; passive moisture protection via a hydrophobic, CO_2_-permeable PTFE membrane; and active moisture protection via a low-power micro-dehumidifier. Our CO_2_ instrumentation performed well and yielded a high-quality dataset that includes signals related to a prescribed fire as well as seasonal and diel cycles. We expect our technology to support underground CO_2_ monitoring in fields where it is already practiced and stimulate its expansion into diverse new fields.

## 1. Introduction

Soil gas probes are distinct from the broader market of atmospheric gas sensors because of the need to survive the humid soil environment while remaining exposed to its pore-space gases. Probes that measure CO_2_ specifically have applications in many different fields, including climate change, agriculture, terrestrial and aquatic ecology, geosciences, industrial sciences, and other fields. In particular, CO_2_ probes are often used to calculate CO_2_ fluxes from soils to the atmosphere; this role is relevant to the tasks of accounting for soil as a source or sink in the overall carbon budget and for monitoring storage of both organic and inorganic carbon in soils. Additionally, in life sciences, CO_2_ probes are increasingly used in partitioning sources of CO_2_ (e.g., autotrophic vs. heterotrophic organisms) from terrestrial ecosystems when paired with eddy covariance towers and/or chamber CO_2_ measurements. These sensors are also used in laboratory and greenhouse experiments to monitor plant growth and CO_2_ uptake.

It is inherently difficult to design a soil gas sensor that is simultaneously exposed to the soil air while also being adequately protected from its high humidity and occasional saturation of the soil. Because of this, scientists interested in monitoring soil CO_2_ with in situ soil probes have suffered from limited selection, high cost, and often poor reliability of off-the-shelf CO_2_ probes. These issues persist even after many years of performance problems and iterations on soil probes. As an indicator of community recognition of the need for different in situ soil CO_2_ sensing options, some researchers have developed their own less-expensive homegrown tools [1,2]; however, these solutions have not been commercialized or seen widespread use.

As an alternative to in situ soil probes, soil gases may be monitored by sensors placed on the surface, connected to either surface chambers or an underground study point using tubing (e.g., [3,4,5]). These measures can (1) directly measure CO_2_ efflux using surface chambers, with the increasing CO_2_ concentration over time equating to soil respiration (R_soil_), or (2) monitor CO_2_ concentration at one or multiple depths underground (depending on if concentration or CO_2_ flux is needed) using buried tubing. Both methods usually include a single above-ground sensor and manifold, and both methods have their own strengths and weaknesses. Compared to in situ soil CO_2_ sensors, chamber methods offer simple installations and measurements of R_soil_. However, they cannot provide high-frequency monitoring of R_soil_; they require manual or mechanical venting to avoid negative feedback on CO_2_ efflux, which is expensive and/or logistically challenging; and they are often connected to more expensive infrared gas analyzer (IRGA) or gas chromatography (GC) sensors. Chambers also lack the ability to determine details of CO_2_ consumption and production at specific soil depths. The inserted tubing connected to a tubing manifold and sensor has the benefit of easier installation and the absence of the need to recover expensive sensors. However, it is necessary to avoid leaks, crimps, and condensation in a network of tubes running through the soil, into a manual or automated gas manifold, and then into the sensor, which is often difficult and requires more frequent maintenance. Furthermore, the gas sensor connected to the tubing must have an air circulation pump, which draws a certain flow rate, and, in finer-textured soils, often creates a strong negative pressure that subsequently samples soil gas from alternate regions of the profile or draws surface air in through preferential flow paths. Finally, like the chamber method, the tubing method is often associated with expensive IRGA or GC analyzers.

In this paper, we describe our new soil CO_2_ logging system (named the “Artemisia” after the genus of sagebrush common in the western US landscapes where we work). The Artemisia employs multiple innovations to enable soil probes to survive the humid soil environment while being feasible to produce affordably at a small scale. We also describe its use in a multi-month campaign, in which it survived extreme soil temperature and moisture conditions, and show recordings exemplifying its data quality.

## 2. Materials and Methods

In this section, we describe the hardware, firmware, software, and field methods associated with the CO_2_ logging system.

### 2.1. Waterproofing

Surviving the humid soil environment while remaining coupled to the soil air is the main design challenge for soil gas sensors. Specifically, our photoacoustic CO_2_ sensor has a maximum relative humidity (RH) specification of 95% to avoid condensation. An inadequately protected soil probe exceeds this limit because of the equilibration with soil air (which, except in dry soil conditions, is near 100% RH) and, in saturated conditions, the ingress of liquid water. We control humidity via multiple active and passive approaches.

Active humidity management consists of a solid-state electrical dehumidifier membrane [6] attached to the probe wall, with a fan inside the probe to provide air circulation. The dehumidifier is a solid polymer electrolyte membrane where, when direct current is applied to it, hydrogen ions are separated from H_2_O molecules in water vapor at the anode (dehumidifying) side, transported to the cathode side, where they react with oxygen in the air to form H_2_O molecules, and discharged into the environment. Together, the fan and dehumidifier draw approximately 53 mA for each probe. In case of power interruption, dehumidification ceases, and the probe interior gradually equilibrates with the environmental humidity until power is restored.

Passive humidity management includes a waterproof, gas-permeable membrane for gas exchange, along with a 3D-printed enclosure whose design follows practices that ensure good waterproofing. These features reduce the ingress of liquid water into the probe and make active dehumidification more effective. Gas exchange between the sensor and environment occurs over a PTFE (“Teflon”) membrane, which is impermeable to liquid water but allows diffusion of CO_2_, water vapor, and other small gas molecules [7]; these useful properties of PTFE have been exploited by previous work on soil gas probes [8]. The PTFE membrane is constructed from commercial-grade PTFE tape placed across the probe opening and physically supported by a 3D-printed screen, and the membrane is secured via compression between a 3D-printed ring that fits against the sensor chassis. O-rings prevent leakage around the PTFE membrane. Commercial-grade PTFE tape has a pore size of ~0.5 μm [9], with typical density between 0.50 and 0.70 g/cm^3^; the Electro-Tape brand PTFE we used has a manufacturer-specified thickness of 0.08 mm.

In addition to the gas exchange surface, waterproofing of the probe enclosure itself is required. However, additive manufacturing (i.e., 3D printing, our preferred manufacturing method at small production scales) normally performs poorly at preventing liquid water ingress. In addition to standard waterproofing practices, like using waterproof cable connectors and rubber o-rings at joints, we employed multiple methods to improve the waterproofing of our additive-manufactured sensor enclosure. We utilized an additive manufacturing method known as fused filament fabrication (FFF) because of its low cost, available materials, and ease by which edits can be made to the part during development. Though FFF 3D printing is well known for its ease of use, it is also known for its poor surface quality due to structural defects created during the printing process, which often limits the application of FFF to prototyping only [10]. 

To ensure our enclosure can prevent liquid water ingress, we needed both an environmentally robust plastic and design principles that reduce surface defects associated with the FFF process.

Acrylonitrile styrene acrylate (ASA) is a robust, UV-resistant thermoplastic widely available for use with FFF 3D printers [11]. We chose a 1.75 mm PolyLite ASA filament with a density of 1.1 g/cm^3^, maximum water absorption of 0.4% by weight, and glass transition temperature of 97.8 °C. Before printing the sensor housing, the filament was dried in a filament dryer at 65 °C for ≥12 h. In practice, this drying process improved the surface finish of our printed models and reduced part warping as the model cooled compared to when we used undried filament.

To complement our material choice, we further improved the waterproofing of the sensor housing by several critical design principles: rounded corners, a 1.2-mm shell thickness, an extrusion flow ratio (k-value) greater than 0.98, and randomized print seams [10]. Rounded edges improve layer adhesion at its corners during printing, which reduces the production of micro-pores. A sensor housing wall thickness of at least 1.2 mm reduces the likelihood of liquid water ingress through micro-pores. Printing enclosures using an extrusion flow ratio (k-value) greater than 0.98 reduces inter-layer voids and improves layer adhesion to reduce micro-pore formation. Finally, seam randomization, where seams are the position where the FFF 3D printer begins the deposition of a new line of plastic for each print wall, reduces the likelihood of liquid-water infiltration through surface micro-pores [10].

To additionally reduce liquid-water ingress through the sensor housing’s outer surface, we inserted each probe into a shallow glass dish of 100% acetone and rotated it to chemically re-flow the entire exterior. The probes were removed from the acetone bath quickly and allowed to dry for 48 h before assembly. This treatment eliminated visible external layer lines inherent to FFF 3D printing that contribute to micro-pore formation [10].

The data logger enclosure also had to be weatherproof and UV-resistant to survive long deployment periods at the ground surface. Because the data logger did not require gas exchange with its surroundings and because the air at the ground surface was less humid and prone to water entry than the soil, adequate protection could be achieved using an off-the-shelf watertight box with penetrations sealed and cables connected via waterproof connectors.

### 2.2. Electronics

**Figure 1:**
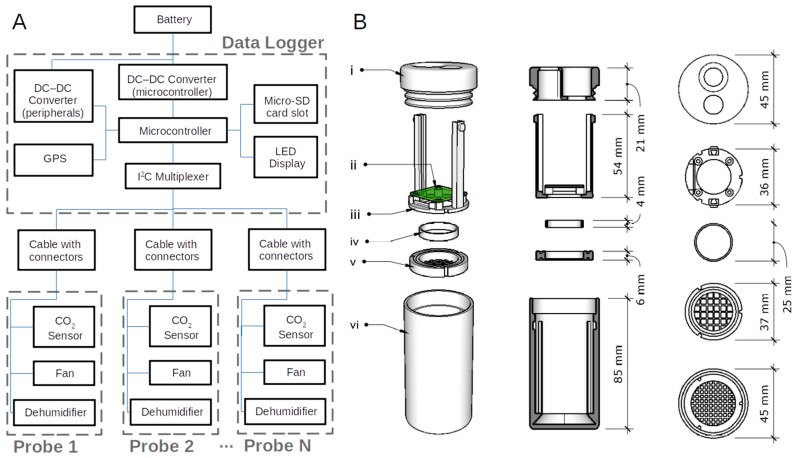
(**A**) Schematic of CO_2_ logging system. An external battery connects to the data logger with a cable; the data logger connects to several sensor probes via cables. (**B**) Components of the probe include (i) cap with openings for active membrane and cable (sealed with O-rings), (ii) sensor circuit board, (iii) rack with lock pins for mounting the sensor circuit board, (iv) ring for attaching PTFE membrane, (v) PTFE membrane screen (sealed with O-rings above and below), and (vi) probe housing.

The CO_2_ logging system consists of a single data logger connected to multiple probes by cables (Figure 1). Each probe includes one SCD41 photoacoustic non-dispersive infrared (NDIR) CO_2_ sensor [12] exposed to soil air via a gas-permeable membrane (described in Section 2). The SCD41 has appropriate specifications for measuring soil CO_2_: it has a wide measurement range of 0–40,000 ppm CO_2_ (able to measure spikes in CO_2_ concentration in extreme events) with an accuracy finer than ±(50 ppm + 5% of the reading) for the 400–5000 ppm range (common for typical soil conditions) and a response time on the order of one minute (much faster than most soil processes that affect CO_2_ concentration). The SCD41 includes built-in temperature/humidity sensors used for on-chip CO_2_ signal compensation. It was also possible to achieve on-chip pressure compensation by programming it with the altitude of data collection; however, we did not actually implement this, reasoning that pressure compensation in post-processing was preferred to adding a configuration step to each deployment and maintenance site visit. Instrument drift is modest over a five-year time scale and can optionally be corrected by built-in recalibration routines using ordinary atmospheric air. The SCD41 also records temperature and humidity (Table 1), which are included in data files and may be treated either simply as state-of-health data or as additional information about the state of the soil. We note that although other CO_2_ sensors have higher accuracy than the SCD41 [13], it is accurate enough for a wide range of applications that can benefit from our technology’s other characteristics.

The data logger includes a Cortex M4 microcontroller, which we selected because it has excellent specifications for our project and is included in development boards in the widely used Arduino family (thereby having access to pre-existing libraries and example code). The Arduino platform of development boards and libraries was already known through the authors’ prior instrumentation development experience to be an efficient way for scientists to develop custom instrumentation [14]; we emphasize the importance of using common, well-tested, well-supported, user-friendly electronics and libraries to eliminate technical obstacles and distractions during development. Compared to other hardware in the Arduino family, including the Arduino Uno R3 and other popular development boards based on the older ATMEGA328P and ATMEGA32u4 microcontrollers, the Cortex M4 (used in the vendor Sparkfun’s Openlog Artemis and Redboard Artemis, for example) has much more memory for programs and data (which simplifies firmware programming by allowing the use of more libraries and reducing the need to optimize for memory) and has much lower power consumption.

The microcontroller communicates with most peripherals using the I2C protocol. Because all sensors have the same I2C device address, an I2C multiplexer was used to let the microcontroller communicate with a single designated sensor at a time. Users benefit from the flexibility provided by long cables to deploy probes up to tens of meters apart as needed; we have found that using shielded cables and a low data rate of 10,000 kbps ensures reliable I2C communication over these long distances. The I2C bus also includes an LED display to report system performance to the user and a GPS for periodically obtaining date and time accurate to the second. The microcontroller connects to a user-accessible micro-SD card slot over an SPI connection. The data logger contains two switching regulators to efficiently reduce power supply voltage to the electronics’ operating voltage; one powers the microcontroller and micro-SD slot, and one powers the peripherals and is controlled by the microcontroller, enabling it to power-cycle peripherals as needed. Both regulators draw power from the same battery cord.

On startup, the microcontroller resets the micro-SD card, I2C multiplexer, and all sensors; communicates to the user that they are responding; and waits for a GPS fix. Upon obtaining a GPS fix, the microcontroller enters the sampling loop, where it waits according to the programmed sample interval (as short as five seconds), reads data from all sensors, writes a new formatted line to the micro-SD card, and then waits again. In each iteration, the microcontroller attempts to reset any unresponsive sensors, and the use of a watchdog timer allows the logger to recover from system-wide errors by resetting itself after blinking an error code to the user. Upon reaching the end of the day (00:00 UTC), the microcontroller resets itself, resulting in a full system restart and a new data file at the beginning of the next day. 

### 2.3. Deployment Methods

**Figure 2:**
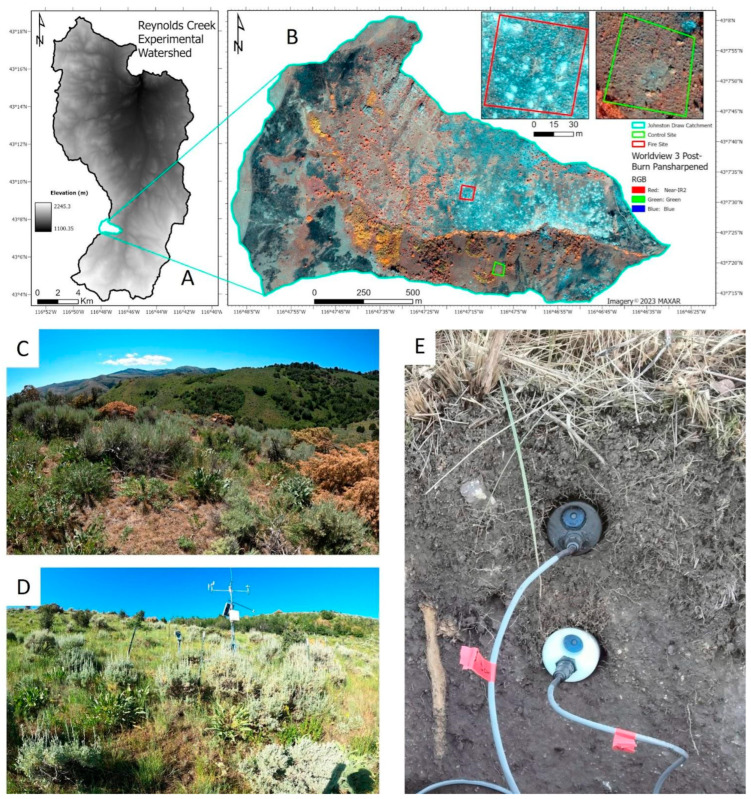
Site information and instrumentation details. (**A**) The test site was located in the Reynolds Creek Experimental Watershed (RCEW), located in southwest Idaho, USA. (**B**) False-color image of the Johnston Draw sub-catchment where CO_2_ probes were tested. The figure is derived from WorldView 3 imagery ~4 days after a prescribed fire; test sites on contrasting aspects are outlined in the sub-catchment. (**C**) Pre-fire vegetation cover for the wetter north-facing site that acted as a burn control. This site had deeper soils, greater water contents, and substantial snowpack from winter. (**D**) Pre-fire vegetation cover for the drier south-facing site that included measures of soil CO_2_ concentration during the prescribed fire. The site had shallow rocky soils, coarser texture, and lower water contents, and it was only intermittently covered by snow. (**E**) Example installation of the CO_2_ probes inserted horizontally into a soil profile prior to backfilling the soil pit.

Probes are installed either by (1) digging a vertical soil profile face, boring a horizontal hole the same diameter as the probe into the face, inserting the probe, and backfilling (e.g., Figure 2E) or (2) by boring a vertical hole to a specific soil depth, lining with conduit, and bracing the probe against the conduit base to form a seal. In our field campaign described in Section 3.2, we used the former method, where sensors are installed underground. Once installed, probes were left undisturbed until the end of the field campaign. Data loggers were left on the surface to maintain GPS reception (for sample timing) and user accessibility for data downloads and maintenance. To begin acquisition, the user simply connects all probe cables, connects power, and watches the LED display to ensure that all sensors are connected properly and that the logger is acquiring data. To end acquisition, the user disconnects power; power should be disconnected when removing or inserting a micro-SD card.

In long campaigns far from mains power, most users power the CO_2_ logging system using a 12-V lead-acid battery connected to a solar panel. Users should select batteries and solar panels capable of meeting the average and peak current draws (Table 1), plus a safety margin, during the least sunny month at their site. The logging system provides all data in real time over a widely supported 115200-8-N-1 serial port for possible use in telemetry or real-time viewing; although we do not expect this feature to be used in most field campaigns, it makes our instrument viable in educational or real-time-monitoring roles.

### 2.4. Rtemisia Package

Data files from the CO_2_ logging system are in plain-text .csv files with a short header, readable in any spreadsheet or scientific computing program. To speed up data processing and visualization, we provide the open-source “Rtemisia” R package [15]. We selected R as the working language, anticipating it to be the most popular data analysis platform among our community of likely users, because it is free and open-source and because it is a natural choice for workflows involving statistical analysis and high-quality plots. Rtemisia includes functions to read files as data frames, merge them, and make routine plots of CO_2_ concentration, temperature, and humidity (showing all probes on the same axes for comparison). It also includes functions to correct sensors’ automated self-calibrations when needed (see Section 2.2).

## 3. Results

### 3.1. Calibration

The CO_2_ sensor we used has accuracy specifications provided on its datasheet, but only for the 400–5000 ppm range. The sensor can measure up to 40,000 ppm, so most of its range is not described by an accuracy specification in its datasheet. We conducted our own calibration to better understand its typical accuracy at high concentrations.

Calibration was conducted on four sensors by inserting the probe into a 3D-printed test stand with an O-ring seal around the exterior of the probe, and standard gas passed through the test stand/sensor headspace at a rate of 1 L/min. Baffles were included in the stand headspace to create turbulent flow and a well-mixed headspace for sensor calibration. The probe’s PTFE membrane was removed to facilitate rapid air mixing, and the calibration was allowed to run for 5 min to completely purge the probe headspace (~100 mL).

Our standard gases included ordinary air with 0 ppm CO_2_ and 10,000 ppm (±2%) CO_2,_ with the remainder being N_2_; standard gas concentrations were measured by Fourier-transform infrared spectroscopy. Both CO_2_ concentrations tested (0 ppm and 10,000 ppm) are outside the range for which a manufacturer’s accuracy specification is available.

**Figure 3:**
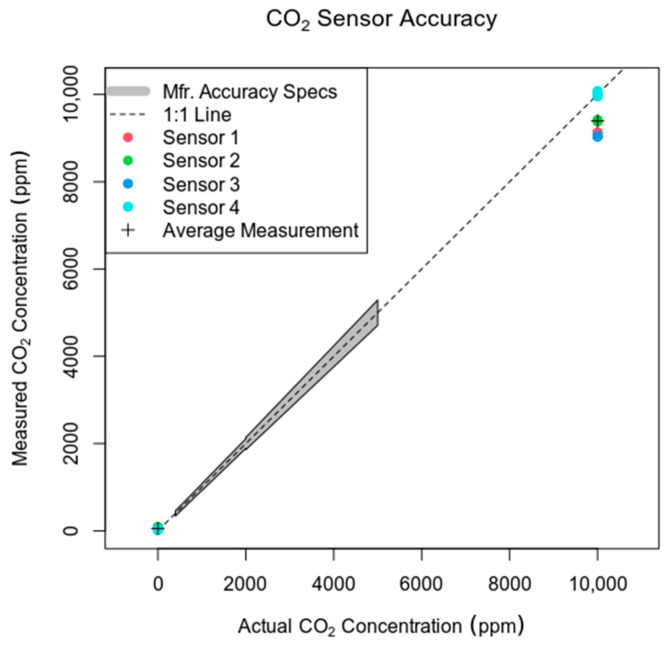
Measured vs. actual CO_2_ concentrations for sensor calibration and correction. The manufacturer’s accuracy specification spans the range of 400–5000 ppm (shaded gray region). When tested at 10,000 ppm, well outside that range, all measurements from the four sensors fell within 10% of the true value, with an average underestimate of 6.1%.

At 10,000 ppm, measurements from all sensors ranged from 9038 ppm to 10,075 ppm, with an average of 9394 ppm (a 6.1% underestimate) (Figure 3). This suggests that high-concentration measurements by our system, using this particular CO_2_ sensor type, may somewhat underestimate the true CO_2_ concentration.

### 3.2. Field Campaign

The probes were installed and tested in an already well-instrumented catchment within the Reynolds Creek Experimental Watershed (RCEW) [16] (Figure 2A) in collaboration with the US Department of Agriculture’s Northwest Watershed Research Center (NWRC). The Johnston Draw catchment (Figure 2B) is 1.8 km^2^, predominantly east–west oriented, and the site of an October 2023 prescribed fire to reduce the expansion of Western Juniper in rangelands. Two sites were selected in June 2023 to test the sensors based on aspect, elevation, vegetation density, soil depth, and moisture regime. In addition, one site was burned during testing, while the other site remained unburned. The burned site (Figure 2C) was sparsely vegetated, south-facing, coarsely textured, and well-drained resulting in lower soil moisture content. The unburned site (Figure 2D) was densely vegetated for rangelands, north-facing, more finely textured because of dust deposition and enhanced weathering, and thus poorly drained, which resulted in greater soil moisture contents. Two replicates were installed at each site in a randomized block design under three different vegetation types: interplant patches, sparsely vegetated and dominated by grasses and forbs; underplant patches located beneath mature Wyoming Big Sagebrush (*Artemisia tridentata* subsp. *wyomingensis*); and underplant patches located beneath felled Western Juniper (*Juniperus occidentalis*) that had been cut in June of 2023. Probes were installed horizontally into a soil pit face (Figure 2E) at two depths per plot (5 and 15 cm depths) for a total of 24 probes. Soil moisture probes (Stevens HydroProbe, Portland, OR, USA) and thermocouples (Omega, K-type, GG-K-30-500, −73 to 482 °C) were installed at the same depths adjacent to the CO_2_ probes to monitor soil conditions and facilitate CO_2_ flux calculations using the flux-gradient method [17].

### 3.3. Field Campaign Results and Instrument Performance

**Figure 4:**
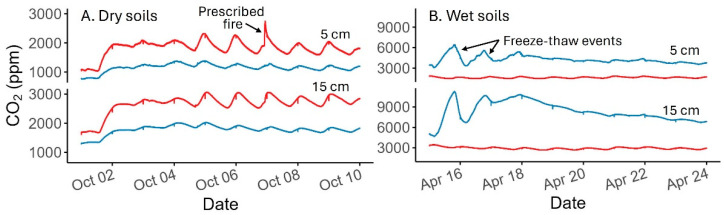
Soil CO_2_ probe data during the testing period. (**A**) Soil CO_2_ concentrations at 5 and 15 cm depths approximately 2 weeks after installation and representative of dry soil conditions. (**B**) Soil CO_2_ concentrations at 5 and 15 cm depths approximately 6 months after installation and representative of wet and cold soil conditions. Red lines are data collected from the hotter and drier south-facing aspect site, which burned on 6 October 2023; blue lines are data collected from the cooler and wetter north-facing aspect site (unburned), which was buried under snow from December to April.

Our instrumentation functioned well over approximately 7 months of operation in a cold desert that included rain and snow, freeze–thaw events, soils at near water holding capacity, and notably hot and cold temperatures during the fire and winter. All four loggers and 24 probes were still operating normally at the end of these 7 months (Figure 4). The malfunctions that did occur were associated with poor GPS reception in the data loggers and due to solar/battery power systems that, until upgraded, provided insufficient winter power at the low-insolation site. No sensors showed any signs of malfunction or failure due to intolerance of the soil environment.

The data loggers in their waterproof boxes were housed in a secondary water-resistant cooler inserted into the soil but not fully buried. Under these conditions, there were no issues with logger operation due to moisture. However, the GPS signal (and, therefore, the ability to write data with correct times) was lost occasionally at the south-facing site during the winter, possibly due to thick wet snow intermittently covering the internal GPS antenna, and was reacquired without user intervention.

Instrumentation at the north-facing slope suffered from nightly power loss and subsequent disconnection in the early winter due to limited insolation on steep terrain and sharing power with a higher-priority permanent meteorological station. The installation of an upgraded photovoltaic system in January 2024 restored consistent power to our CO_2_ monitoring instruments at this site. Despite the CO_2_ probes’ active dehumidifiers not being able to operate during the power outage, the probe function appeared to be uncompromised by sitting in the humid soil environment without power for ~2 months (Figure 4B).

**Figure 5:**
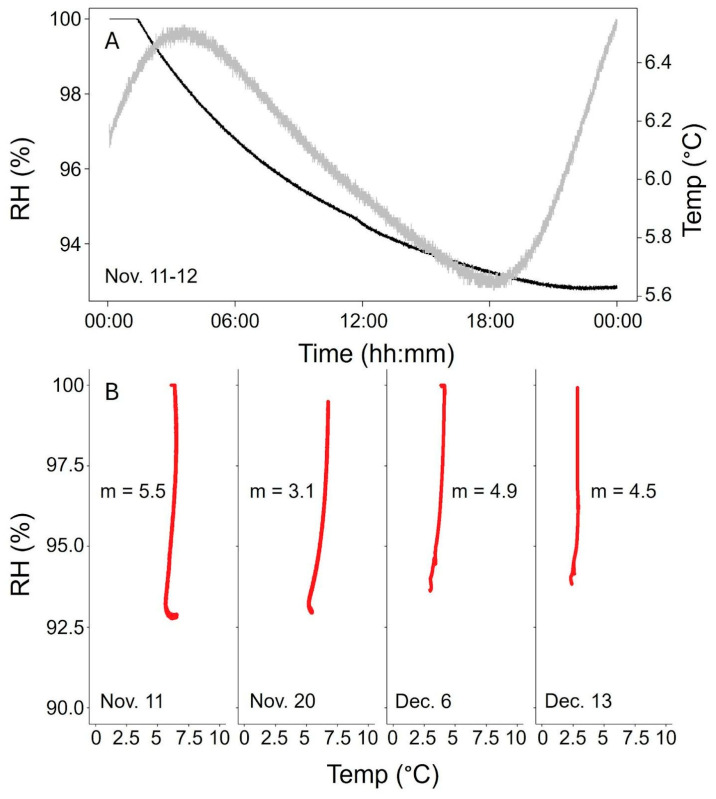
Assessment of probe active membrane RH control as measured by IR sensor. (**A**) Plot of change in RH (black) and soil temperature (gray) at 15 cm depth following precipitation event on November 10. (**B**) Regression of temperature vs. RH for four example post-precipitation events during 2023 (m = linear slope of temperature–RH relationship).

In the soil environment, the soil moisture and high humidity pose a substantial challenge to electronics. The IR sensor selected for the initial CO_2_ probe tests also included integrated sensors for RH (±9% accuracy) and temperature (±1.5 °C accuracy); therefore, our long-term dataset served as a test of the probes’ active and passive moisture protection. In four rain events in November–December 2023, when the soil was generally cold and dry, RH in the probes rose immediately after the rain as expected and dropped over the following hours despite temperature decreasing as well (Figure 5). Because RH depends inversely on temperature, assuming no moisture is added or removed, we attribute the decreasing RH in the probes to the active dehumidifier. We note that in the colder, wetter soil conditions later in the winter, RH in the probes was generally higher than in the fall and spring (suggesting that further adjustments to our moisture protection system may be warranted to adapt our probes to a wider range of climate settings), although the sensors continued operating with no signs of malfunction.

## 4. Discussion

Our field campaign demonstrates the ruggedness and utility of our CO_2_ logging system for in situ soil monitoring for periods of at least several months, surviving the environmental challenges of moisture and temperature extremes and yielding a high-quality dataset showing diel and seasonal CO_2_ fluctuations. Its performance and specifications demonstrate its suitability for monitoring underground CO_2_ production and movement in diverse scientific and agricultural applications. We expect our rugged, low-cost technology to help broaden the use of in situ gas monitoring into new fields, such as carbon sequestration, magmatic gas hazards, and fugitive landfill gases.

Future improvements are expected to focus on ease of manufacture, overall professionalism, and user experience to stimulate distribution and use outside of our group. In particular, we will create a rugged enclosure for the data logger that enables easy user access to batteries, memory card, and LED display while protecting electronics even when the user opens the enclosure. We will improve the LED display to be easily intuitive even to novice users (e.g., indicating when data are being acquired and alerts of disconnected or unresponsive sensors, missing or unresponsive memory card, and lack of GPS reception) while providing advanced diagnostics that can be used in more sophisticated troubleshooting or repair (e.g., confirming functionality of subsystems). We will continue testing and applying new approaches for sensor-probe waterproofing for protection against extreme soil saturation conditions. Furthermore, our calibration at high CO_2_ concentration (outside the range for the manufacturer’s accuracy specifications) shows that further sensor-level calibration can improve accuracy at concentrations above 5000 ppm (which did occur temporarily in our field experiment). Finally, we will extend our technology’s reach by evaluating and integrating sensors for methane (e.g., for measuring the role of soil uptake in the global methane budget [18]) and possibly other gas types into our waterproof probes.

## 5. Conclusions

We have developed a soil CO_2_ logging system consisting of a user-accessible data logger connected to several NDIR sensors, each enclosed in a buried gas-permeable probe. The probes use 3D-printed housings designed for waterproofing, passive hydrophobic membranes, and active solid-state dehumidification to achieve adequate moisture protection in continuously humid and wet soils. A data logger based on the popular Arduino system connects to multiple sensors, manages their activity, and writes data to a disk. We recorded a high-quality dataset using four of our logging systems (each with six sensors) during a several-month-long field campaign that included a prescribed fire and overwintering (thereby creating both temperature and moisture extremes). Our results show expected seasonal and diel cycles and further demonstrate the variability of CO_2_ concentration by depth of burial and vegetation cover. The monitoring of sensor probe humidity confirms the performance of our active dehumidification system. We expect our soil CO_2_ monitoring system to expand in situ gas monitoring in geochemistry, soil ecology, agriculture, and other fields where it is already used and to support its adoption in innovative applications.

## 6. Patents

US provisional patent application 63/690,910: Soil Gas Logging System (2024) [19].

## Figures and Tables

**Table 1:** Estimated specifications of the Artemisia CO_2_ logging system. Sensor accuracy and repeatability specifications are drawn from the sensor vendor’s datasheet [12]. Peak and average current draws are estimated from the sensor vendor’s datasheet and measurements of other components’ current draws, and we assume that one logger is powering six sensors.

Specification	Value
Safe Data Logger Temperature Range	−40 to 85 °C
Safe Power Supply Voltage	<36 V
CO_2_ Accuracy (400–5000 ppm)	±(40 ppm + 5% of reading) or better
CO_2_ Repeatability	±10 ppm
Humidity Accuracy (−10 to 60 °C, 0–100% RH)	±9% RH or better
Humidity Repeatability	±0.4% RH
Temperature Accuracy (−10 to 60 °C)	±1.5 °C or better
Temperature Repeatability	±0.1 °C
Current Draw (Averaged Over Time)	158 mA
Current Draw (Peak)	548 mA
Real-time Output Format	Serial (115200-8-N-1); over USB or TTL UART

## Data Availability

No new data were created or analyzed in this study. Data sharing is not applicable to this article.

## References

[1] Blackstock J.M., Covington M.D., Perne M., Myre J.M. (2019). Monitoring atmospheric, soil, and dissolved CO_2_ using a low-cost, Arduino monitoring platform (CO_2_-LAMP): Theory, fabrication, and operation. Front. Earth Sci..

[2] Perez Rojas Y.T. (2023). Measuring soil CO_2_ emissions with air-quality sensors. Nat. Rev. Earth Environ..

[3] Brecheisen Z.S., Cook C.W., Heine P.R., Ryang J., Richter D.d.B. (2019). Development and deployment of a field-portable soil O_2_ and CO_2_ gas analyzer and sampler. PLoS ONE.

[4] Parkin T.B., Venterea R.T., Follett R.F. (2010). Sampling Protocols. Chapter 3. Chamber-Based Trace Gas Flux Measurements. Sampling Protocols.

[5] Rochette P., Eriksen-Hamel N. (2008). Chamber Measurements of Soil Nitrous Oxide Flux: Are Absolute Values Reliable?. Soil Sci. Soc. Am. J..

[6] Westside International Electrolysis Type Dehumidifying Element. https://www.micro-dehumidifier.com/wp-content/uploads/2017/04/ROSAHL_English_catalogue-2014.pdf.

[7] Parsons B.A., Smith O.L., Chae M., Dragojlovic V. (2015). Properties of PTFE tape as a semipermeable membrane in fluorous reactions. Beilstein J. Org. Chem..

[8] Gil-Loaiza J., Roscioli J.R., Shorter J.H., Volkmann T.H.M., Ng W.R., Krechmer J.E., Meredith L.K. (2022). Versatile soil gas concentration and isotope monitoring: Optimization and integration of novel soil gas probes with online trace gas detection. Biogeosciences.

[9] Oberg K.A., Palleros D.R. (1995). Teflon tape as a sample support for IR spectroscopy. J. Chem. Educ..

[10] Gordeev E.G., Galushko A.S., Ananikov V.P. (2018). Improvement of quality of 3D printed objects by elimination of microscopic structural defects in fused deposition modeling. PLoS ONE.

[11] Sedlak J., Joska Z., Jansky J., Zouhar J., Kolomy S., Slany M., Svasta A., Jirousek J. (2023). Analysis of the mechanical properties of 3D-printed plastic samples subjected to selected degradation effects. Materials.

[12] Sensirion (2023). SCD4x: Breaking the Size Barrier in CO_2_ Sensing (Version 1.5). https://sensirion.com/media/documents/48C4B7FB/64C134E7/Sensirion_SCD4x_Datasheet.pdf.

[13] Romanak K.D., Bomse D.S. (2020). Field assessment of sensor technology for environmental monitoring using a process-based soil gas method at geologic CO_2_ storage sites. Int. J. Greenh. Gas Control.

[14] Anderson J.F., Johnson J.B., Bowman D.C., Ronan T.J. (2018). The Gem infrasound logger and custom-built instrumentation. Seismol. Res. Lett..

[15] Rtemisia: R Package to Read Data and Make Routine Plots for the Rtemisia CO_2_ Logging System. https://github.com/ajakef/Rtemisia.

[16] Seyfried M., Lohse K., Marks D., Flerchinger G., Pierson F., Holbrook W.S. (2018). Reynolds Creek Experimental Watershed and Critical Zone Observatory. Vadose Zone J..

[17] Verma A.K., Kelleners T.J. (2012). Depthwise Carbon Dioxide Production and Transport in a Rangeland Soil. Soil Sci. Soc. Am. J..

[18] Rosentreter J.A., Alcott L., Maavara T., Sun X., Zhou Y., Planavsky N.J., Raymond P.A. (2024). Revisiting the global methane cycle through expert opinion. Earth’s Future.

[19] Anderson J.F., Huber D.H., Walsh O.A. (2024). Soil Gas Logging System. U.S. Provisional Patent Application.

